# Diphtheria-Tetanus-Polio, Measles-Mumps-Rubella, and Hepatitis B Vaccination Coverage and Associated Factors among Homeless Children in the Paris Region in 2013: Results from the ENFAMS Survey

**DOI:** 10.3390/ijerph17082854

**Published:** 2020-04-21

**Authors:** Samreen Mansor-Lefebvre, Yann Le Strat, Anne Bernadou, Nicolas Vignier, Jean-Paul Guthmann, Amandine Arnaud, Daniel Lévy-Bruhl, Stéphanie Vandentorren

**Affiliations:** 1 Santé Publique France, French National Public Health Agency, F-94415 Saint-Maurice, France; sammansor@yahoo.dk (S.M.-L.); Yann.LESTRAT@santepubliquefrance.fr (Y.L.S.); anne.bernadou@santepubliquefrance.fr (A.B.); jean-paul.guthmann@santepubliquefrance.fr (J.-P.G.); Daniel.LEVY-BRUHL@santepubliquefrance.fr (D.L.-B.); 2 Ecoles des Hautes Etudes en santé Publique, 35043 Rennes, France; 3 European Programme for Intervention Epidemiology Training (EPIET), European Centre for Disease Prevention and Control (ECDC), 169 73 Solna, Sweden; 4 Department of Social Epidemiology, Sorbonne Université, Inserm, Institut Pierre Louis d’Epidémiologie et de Santé Publique (IPLESP), F75012 Paris, France; vigniernicolas@yahoo.fr; 5 Department of Infectious Disease and Tropical Medicine, Groupe Hospitalier Sud Île de France, 77000 Melun, France; 6 Institut Convergences et Migration, 93300 Aubervilliers, France; 7 Observatoire du Samusocial de Paris, 75012 Paris, France; a.arnaud@samusocial-75.fr

**Keywords:** immunization, vaccination coverage, children, homeless

## Abstract

*Background*: The number of homeless families has increased considerably since the 1990s in France. We aimed to estimate the homeless children vaccination coverage (VC) for diphtheria, tetanus, polio, measles–mumps–rubella and hepatitis B and identify factors associated with insufficient VC according to birthplace. *Methods*: A cross-sectional survey was conducted among homeless shelter families in the greater Paris area. A nurse conducted face-to-face interviews and collected vaccination records. We analyzed factors associated with insufficient VC, stratified by birthplace and vaccine, using robust Poisson regression. *Results*: The study included 214 children born in France and 236 born outside France. VC in French-born homeless children was high (>90% at 24 months for most vaccinations) and similar to levels observed in the general population, whereas VC in those born outside France was low (<50% at 24 months for all vaccines). Factors significantly associated with insufficient VC among children born outside France were age, parents with French-language difficulties, and changing residence at least twice in the previous year. Children in contact with the healthcare system at least once in the previous year had significantly higher VC, irrespective of vaccine and birthplace. *Conclusion*: Special attention should be paid to homeless children born outside France, with recent European and French recommendations confirming the need for catch-up vaccination in children with undocumented VC.

## 1. Introduction

In Europe—and France is no exception—families represent the fastest growing segment of the homeless population, particularly in urban areas [1]. This development dates back to the end of the 1990s [2]. In 2010, the homeless emergency services in Paris sheltered more families than single people: more than 11,000 parents and children were accommodated, corresponding to a 300% increase over the previous 10 years [3]. Given this context, in 2013, the *Observatoire du Samu Social de Paris* launched the ENFAMS survey (*Enfants et familles sans logement*), which aimed to describe the health and living conditions of homeless families in the greater Paris area [4]. In the Paris region, the number of sheltered families was estimated at 10,280, which corresponds to an estimated 17,660 children aged under 12 years [4].

A number of studies—mainly from the US—have highlighted the detrimental effects of homelessness on health [5]. Congregate living conditions are a well-known predisposing factor for infectious diseases [6]. Only a small number of recent studies have focused on the effect of homelessness on children’s health [7]. Some have underlined the poorer overall health of homeless children and their higher prevalence of vaccine-preventable diseases compared to children in the general population [8,9,10]. Unstable housing prospects may complicate a successful vaccination program for homeless children [11]. These results contrast with some studies in the US, which point to similarly low levels of vaccination coverage (VC) in homeless children and the general population (with a few age-related differences). However, these studies did not differentiate between early and late vaccination [12]. Young children represent an increasingly greater proportion of the homeless population [13]. With regards to France, few studies have focused on VC in homeless children [14].

According to the French national vaccination program, children should receive most vaccinations by the age of 2 years: three doses (two primary and one booster) of diphtheria, tetanus and polio (DT-IPV), pertussis, Hemophilus influenzae type b (Hib) and pneumococcal vaccines; three doses of the hepatitis B (HepB) vaccine; and two doses of measles–mumps–rubella (MMR) vaccine. Given that herd immunity requires a 95% VC rate for DT-IPV, MMR and HepB, national DT-IPV VC in 2-year-olds is high (96.1% in 2016) [15]. Conversely, VC for MMR and HepB is insufficient, which may explain the series of measles outbreaks in France since 2008 [16].

The first objective of this study was therefore to describe VC for DT-IPV, MMR and HepB in homeless children in the greater Paris area and make comparisons with children in the general population. The second objective was to identify factors associated with insufficient VC in children according to their birthplace.

## 2. Materials and Methods

### 2.1. Study Population

ENFAMS was a large cross-sectional survey conducted in the greater Paris area (Ile-de-France) in 2013. Using time-location sampling [17], it comprised interviews with a representative random sample of families sheltering in emergency centers, long-term rehabilitation centers, social hotels and centers for asylum seekers. A three-stage random sampling design was employed as follows: stage 1: accommodation services were randomly selected; stage 2: families were randomly chosen from a list of residents at each of the selected shelters; stage 3: one child under 13 years of age was randomly selected for each family. We registered 796 accommodation services in the Paris region. Among the 796 services, 251 were randomly selected, 237 contacted and 193 finally visited. The service participation rate was 81%: We sampled 801 homeless families. Family participation rate was 79% [4].

An eligible family was defined as comprising at least one parent (>18 years old) who had at least one child under 13 years of age and who spoke one of the 17 languages included in the survey [4].

Only one parent (preferably the mother) was interviewed by multilingual interviewers using face-to-face standardized questionnaires. A trained nurse examined the children and collected data from their medical records/vaccination cards. For the present substudy, we only included children aged 2 to 13 years whose parents could provide a written record of their vaccinations. No individual electronic vaccination registries were available.

### 2.2. Measures

#### 2.2.1. Insufficient Vaccination Coverage

We focused our analyses on DT-IPV (three doses), MMR (two doses) and HepB (three doses), all monitored by *Santé publique France* (French public health agency), as we aimed to compare them to data produced by the national French vaccination program. Moreover, we chose DT-IPV as a proxy for pertussis and Hib VC, as the multivalent vaccine is used almost exclusively. We chose not to investigate pneumococcus VC, as it is too recent and does not concern the oldest birth cohorts.

French health personnel are required by law to fill in patient health records. The related data for the greater Paris area were available for the 2000–2009 birth cohorts (unpublished Direction of Research, Studies, Evaluation and Statistics data 2000–2003; from 2004 onwards, data available on *Santé publique France* website). VC was estimated with respect to the existing French vaccination recommendations at the time of the survey (2013) [18]. We chose to make comparisons with the French general population of children aged 24 months, the age at which children should have completed vaccination. As the recommendations for two-dose MMR vaccination changed in France in 2005 (second vaccination dose at 13–24 months instead of 6 years), only one-dose MMR VC in homeless children at 2 years of age was compared to the general population. VC was defined as the percentage of children with proof of vaccination from among all children included in the analysis.

For the second objective, the study outcome was insufficient VC for a given vaccination. This was defined as either never receiving the vaccination or not completing all the age-specific doses. We measured VC according to the age of the child.

#### 2.2.2. Covariates: Sociodemographic Characteristics

For child-related data, we included the following variables based on the hypotheses of potential associations agreed by the investigating team after a literature review [15,19]: gender, age, country of birth (for children born outside France), school attendance, and contacts with the healthcare system in the previous year. For parent-related data, we included age, country of birth (for children born in France), family structure, family members in the greater Paris area, educational level, family income, health insurance, number of changes of residence in the previous year, problems with understanding/speaking/reading/writing French, and average length of time living in France (for children born outside France).

### 2.3. Statistical Analysis

Descriptive and regression models took into account various elements of the sampling design (i.e., sampling weights, stratification and sampling units).

Descriptive analyses were performed to assess the characteristics of the study population. VC was estimated and stratified according to the birthplace of the child (born in France versus born outside France). We also analyzed VC according to birthyear in order to investigate trends over time. Birthyears were grouped as follows: 2000–2001, 2002–2003, 2004–2005, 2006–2007, 2008–2009 and 2010–2011.

We used robust Poisson regression to obtain prevalence ratio estimates [20]. Robust Poisson regression analyses were performed to identify factors associated with insufficient coverage and stratified according to birthplace. A robust Poisson regression model was used to assess the crude association between each covariate and insufficient VC for each vaccination. Covariate associations with a *p* value ≤ 0.15 were selected for the multivariate model. Manual backward stepwise variable selection was used to fit the final multivariate model, which retained all covariates with a statistically significant (*p* ≤ 0.05) association with the outcome of interest.

The statistical software package STATA 14.2 (Stata Corp, College Station, Texas, USA) was used for our analysis.

## 3. Results

**Figure 1:**
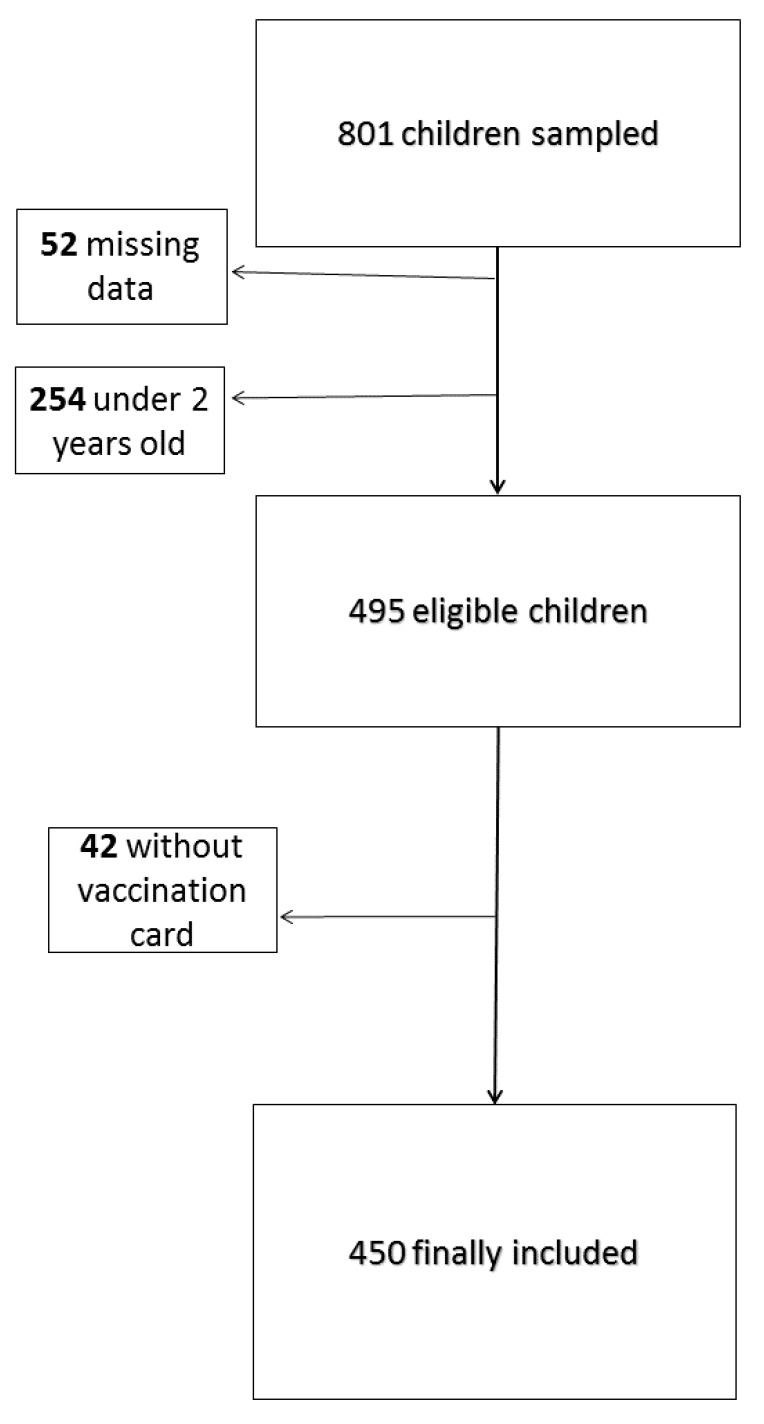
Flow chart.

Of the 801 children initially sampled, 450 met the inclusion criteria (214 and 236 born in and outside France, respectively). Overall, 52 children were not included due to missing data (country of birth or health information), and another 254 children because they were aged under 2 years. Among the 495 eligible children, 42 were excluded from the analysis because they did not have a vaccination record (7 and 33 born in and outside France, respectively) [Figure 1]. The excluded children were more likely to be girls, from European countries, not attending school, and from a low-income family, while they were less likely to be covered by health insurance.

### 3.1. Sociodemographic Characteristics of the Population

Children born in France were younger: 66.1% were aged under 6 years versus 45.2% of children born outside France (Table 1).

Children born outside France were predominantly from Eastern Europe and Africa. Most children had been in contact with the healthcare system in the previous year, especially children born in France. Over 95% of parents were born outside France. Almost half of the parents of children born outside France had difficulties with the French language. The parents of children born outside France were also more likely not to have health insurance (27.4% vs. 9.2% among children born in France). The number of changes of residence in the previous year was higher among children born outside France (36.5% vs. 20.9%).

### 3.2. Vaccination Coverage of Homeless Children

Overall, VC in homeless children was insufficient for all the vaccinations under consideration and lower than that observed in the general population (Table 2).

However, the analysis by birthplace showed higher VC in children born in France (close to 70% for HepB and above 90% for DT-IPV and MMR) than those born outside France (under 50% for all vaccinations). As indicated by the VC ratio, the probability of being vaccinated for a child born in France was between 2.1 and 2.8 times higher than those born outside France depending on the vaccination (*p* < 0.001 for all comparisons).

For DT-IPV, the proportion of children with up-to-date vaccinations increased substantially with age in every birth cohort of children born in France, being close to 100% at 24 months of age (except for the 2010–2011 cohort: 94.0%), thus reflecting the rates observed in the general population of the greater Paris area (average coverage at 24 months of age for the 2000–2009 period: 99.1%). For children born outside France, in every birth cohort, even if these VC proportions tended to increase after 24 months, they were still much lower than those for children born in France (<70% in every birth cohort).

**Figure 2:**
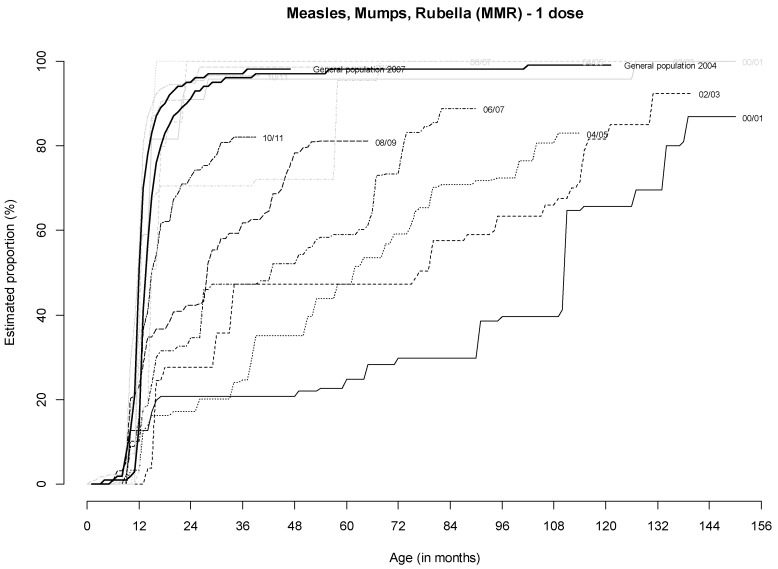
Estimated cumulative proportion of children receiving one dose of MMR according to age (in months) and birthplace in France (gray curves) or outside France (black curves) and according to birth cohort: 2000/01 (solid lines), 2002/03 (dashed lines), 2004/05 (dotted lines), 2006/07 (dot-dash lines), 2008/09 (long-dash lines), 2010/11 (two-dash lines). Note: each point corresponds to the proportion of vaccinated children at a given age.

For MMR (one dose), VC in homeless children born in France was comparable with that in the general population in greater Paris (Table 2). Vaccination coverage was strikingly low at 24 months of age for children born outside France and varied greatly for each birth cohort (Figure 2). However, it increased with age, being up to 80% for some birth cohorts. Only children born in 2010–2011 had a higher VC at 24 months of age (72.4%).

**Figure 3:**
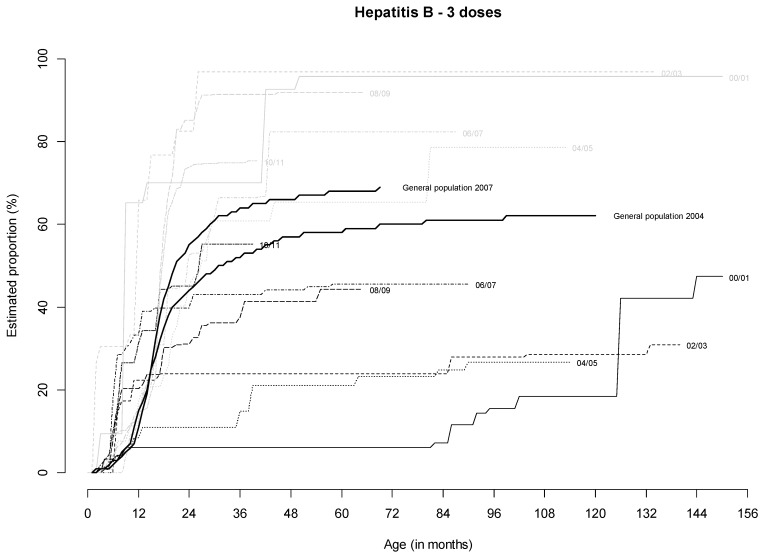
Estimated cumulative proportion of children receiving three doses of hepatitis B according to age (in months) and birthplace in France (gray curves) or outside France (black curves) and according to birth cohort: 2000/01 (solid lines), 2002/03 (dashed lines), 2004/05 (dotted lines), 2006/07 (dot-dash lines), 2008/09 (long-dash lines), 2010/11 (two-dash lines). Note: each point corresponds to the proportion of vaccinated children at a given age.

For HepB (Figure 3), VC in children born in France was close to 70% compared to less than 30% in those born outside France. Moreover, VC in children born in France was higher than that observed in the general population for 2001–2002 (70.0% vs. 37.8%) and 2002–2003 (82.2% vs. 44.5%). Only children in the general population born in 2004–2005 had a higher VC than homeless children born in France from the same birth cohort (55.2% vs. 45.4%)

### 3.3. Factors Associated with Insufficient Vaccination Coverage for DT-IPV, MMR and HepB

#### 3.3.1. Children Born in France

For DT-IPV, children aged 6 to 9 years had a significantly higher risk of being insufficiently immunized than those under 6 years of age (Table 3). Children with a parent born outside France also had a higher risk of being insufficiently immunized. Conversely, children who had been in contact with the healthcare system at least once in the previous year and those who had other family members in the greater Paris area had a significantly lower risk of being insufficiently immunized.

For MMR, children with a parent born outside France had a significantly higher risk of being insufficiently immunized. However, children who attended school, had been in contact with the healthcare system at least once in the previous year and had health insurance had a significantly lower risk of being insufficiently immunized.

For HepB, girls had a significantly lower risk of being insufficiently immunized. Children attending school also had a significantly lower risk of being insufficiently immunized.

#### 3.3.2. Children Born Outside France

For DT-IPV, children aged 6 to 9 years had a significantly higher risk of being insufficiently immunized than those under 6 years of age (Table 4). Children who had a parent with French-language difficulties and those who had changed residence at least twice in the previous year also had a higher risk of being insufficiently immunized. Conversely, children who had been in contact with the healthcare system at least once in the previous year had a significantly lower risk of being insufficiently immunized.

For MMR, as observed for DT-IPV, children born outside France who had a parent with French-language difficulties and those who had changed residence at least twice in the previous year also had a higher risk of being insufficiently immunized. Nevertheless, children who had been in contact with the healthcare system at least once in the previous year and those who had health insurance had a significantly lower risk of being insufficiently immunized. Furthermore, the risk of being insufficiently immunized was significantly lower when the parent had a primary or middle school educational level compared to those with none.

For HepB, children aged 6 to 9 years had a significantly higher risk of being insufficiently immunized than those under 6 years of age. Children who had been in contact with the healthcare system at least once in the previous last year and those whose monthly household income was at least 500 euros had a significantly lower risk of being insufficiently immunized.

## 4. Discussion

Overall, our study showed that for homeless children born in France, VC levels at 24 months of age were higher for HepB and quite comparable for DT-IPV and MMR compared to those found in the general population (which are specifically “satisfactory” for DT-IPV and “insufficient” for MMR and HepB). Nevertheless, homeless children born outside France had considerably lower VC than their French-born counterparts. Children born outside France arrive in the country with incomplete VC because of the different vaccine schedules in other countries, as noted by several studies [21]. Despite the World Health Organization’s introduction of a global standardized childhood vaccination program known as the Expanded Program on Immunization (EPI) several decades ago, the recommendations of individual countries are still based on their own national policies, which reflect disease prevalence/burden of disease, health policies and economic factors. A lower VC may also result from a country introducing a specific vaccination program later than other countries. The low HepB VC observed in our study is a worrying trend, since this vaccine is part of the vaccine schedule in all the countries of origin included our study [22,23]. However, in some cases, populations may also be immunized naturally, meaning that vaccination is no longer relevant. Screening should therefore be performed before HepB vaccination in children coming from an endemic country [24]. Children aged 6 to 9 years had a greater risk of being insufficiently vaccinated compared to those under 6 years, probably because of the catch-up vaccination performed before 6 years by maternal and infant centers in France. The number of times that families changed residence in the previous year was a significant predictor of insufficient VC for DT-IPV and MMR in homeless children born outside France. This could be explained by the fact that most of our study population were migrants, living in social hotels with poor levels of housing comfort and limited services; most of them therefore experienced housing instability. These living conditions have a dramatic impact on health in terms of food insecurity and mental health as described previously [25,26], while women encounter many barriers to accessing health services such as the cost, language, transportation and discrimination or because of other priorities. As homeless women are regularly relocated from shelter to shelter (depending on the available facilities in the chronically under-resourced greater Paris area), primary care professionals must be better informed and aware that every contact with the healthcare services is an opportunity to check whether a child’s VC is up to date. Structures specialized in providing free healthcare to the homeless tend to focus more on treating acute diseases than preventing infectious diseases and in this respect, they could be deprived of vaccines.

In France, health professionals initiate the organization of primary care. Infants and children are mainly cared for by GPs and pediatricians. Parents must pay the non-reimbursed portion of childhood vaccines at the pharmacy, except for those with supplementary health insurance. Most homeless families, however, do not have supplementary health insurance. In this context, it is necessary to improve access to immunization by making vaccines free and available in health facilities [27,28] and thus reducing missed opportunities to immunize children during their contacts with primary healthcare professionals.

Moreover, given the lack of knowledge about how to implement catch-up vaccinations in the absence of prior VC information, some health professionals may be reluctant to intervene. However, all opportunities must be seized.

Our findings indicate that although most homeless children are seen by healthcare professionals; they were not detected as insufficiently vaccinated. Another plausible explanation for the insufficient VC could be language and cultural barriers [29], as identified in our regression analyses. We hypothesized that cultural mediators are not always present, so contacts with the healthcare system in France may sometimes occur in the French language, while prescriptions, referrals and even personal child health records are all written in French. We also hypothesized that even if the importance of vaccination is communicated to parent(s) by medical professionals, this recommendation may be lost or even misunderstood due to the language barriers. We found that 95% of children had at least one parent born outside France and almost half of the parents had difficulties with the French language. This was confirmed by our multivariate analysis for DT-IPV and MMR, where among children born outside France, the parent´s lack of understanding and poor oral skills in French were associated with insufficient VC. However, this was not found for HepB, which may be due to the lack of statistical power in this model. Interestingly, 73.3% of children aged over 3 years attended school (*n* = 330), which may represent an opportunity to improve the planning of vaccination strategies among homeless children. More specifically, given the high proportion of children in our study who were in contact with the healthcare system in the previous year and who attended school, schools could serve as ideal locations to refer children for child health assessments (including VC). In France, health-related data (including vaccinations) are systematically gathered for children in maternal and child protection services (at 9 and 24 months of age, respectively), in preschools and primary schools (at 6 and 11 years of age, respectively) and in ninth grade (15 years). This allows the identification of children who are not up to date with their vaccination. Free vaccinations could be proposed as a possible way to improve the existing system in which school doctors do not provide or recommend vaccinations and in which vaccinations are only available at dedicated structures (maternal and child health centers and free vaccination centers, with disparities across the French territory) or in the context of specific interventions (for example, during the recent measles epidemics). Although the objective is for these centers to adequately cover the whole territory, they are currently deployed and financed in an unequal way, which limits access.

In our study, an increase in VC was observed with increasing age, but it was still insufficient for children born outside France. HepB VC was better in French-born homeless children than in the general population. This probably reflects physicians’ perception of the higher risk among these children who often come from countries with high HepB endemicity and who are more likely to be exposed to social insecurity. Another explanation could be the higher prevalence of maternal chronic HepB, which justified child immunization at birth.

Our study has several limitations. First, as it is cross-sectional and not cohort-based, causality cannot be established. Specifically, we chose to only include children with a personal child health record/vaccination card. This choice was made because, in addition to racial/ethnic differences, overreporting is known to be more frequent among low-income households [30]. Given the characteristics of the minority of children without health records, the observed VC may have been overestimated. However, the French National Health Authority recommends systematic catch-up vaccinations in the absence of vaccine evidence. Following this logic, the VC observed in our study would be even lower if these children were taken into account.

Second, insufficient VC in children born outside France must be interpreted with caution. More specifically, children’s VC before arriving in France could have met the vaccination recommendations existing in their country of origin. In this case, catch-up vaccinations should have been implemented shortly after the child’s arrival in France. Yet our study showed that this occurs very slowly and not on a systematic basis. Furthermore, records about previous vaccinations in the countries of origin may be missing from the submitted vaccination documents. It is therefore possible that the observed differences are related to the suboptimal recording of vaccinations before the child’s arrival in France. Although this should not limit children’s catch-up vaccinations, it strengthens the likelihood that the proportion of protected children is probably higher than the observed coverage.

## 5. Conclusions

Despite these limitations, our study shows that homeless children in France are inadequately immunized. Increasing the VC rate is therefore crucial to ensure better health. Official catch-up vaccination guidelines were recently created for children whose pre-emigration vaccination history is not documented [31,32]. Public health interventions that show a cultural understanding are also needed to target homeless children. Moreover, our results point out the high likelihood of missed immunization opportunities despite the high percentage of children who consulted healthcare professionals in the previous 12 months. This could be explained by the fact that these children have many medical needs and that vaccination status is not prioritized [33]. Furthermore, the presence of acute infectious diseases with or without fever at the time of medical consultation is often a hindrance to the implementation of catch-up vaccinations despite the existence of recommendations emphasizing vaccination without delay [34].

Different strategies are needed to increase the effectiveness of programs for improved CV. School-based vaccinations do not capture preschool children and on-site vaccinations at housing facilities, while ensuring minimal vaccination, may pose challenges for follow-up.

Qualitative studies focusing on structural and individual factors are needed to better highlight and understand the reasons behind suboptimal VC in this population. Nevertheless, these data suggest that foreign-born children in France are not as well vaccinated as other children. Our results point to the type of barriers to vaccination access among these children such as language barriers, economic status and residential instability. Special attention should therefore be given to this population to ensure more accessible vaccination.

## Figures and Tables

**Table 1:** Characteristics of homeless children in the sample (*n* = 450) and estimated as a percentage of characteristics according to birthplace.

	Children Born in France			Children Born outside France			Comparison between Children Born in and Outside France
	Sample Size. n	Estimated Proportion (%)	95% CI	Sample Size. n	Estimated Proportion (%)	95% CI	*p* Value
**Gender**	**214**			**236**			0.9392
Male	105	48.9	40.3–57.5	111	49.3	41.2–57.5	
Female	109	51.1	42.5–59.7	125	50.7	42.5–58.8	
**Birthplace**	**214**			**236**			
France	214	100					
Europe (except France)				100	46.7	38.2–55.4	
Africa				93	40.1	31.7–49.1	
Asia				40	12.3	8.7–17.3	
America				3	0.9	0.2–3.2	
**Age (years)**	**214**			**236**			0.0008
2–5	153	66.1	55.9–75.0	110	45.2	38.1–52.6	
6–9	48	27.0	19.3–36.4	80	34.0	26.8–42.1	
≥10	13	6.9	3.5–13.2	46	20.7	14.2–29.2	
**School attendance**	**159**			**217**			0.0005
No	8	3.1	1.3–7.2	38	16.2	11.0–23.3	
Yes	151	96.9	92.8–98.7	179	83.8	76.7–89.0	
**Child in contact with healthcare at least once in previous year**	**211**			**236**			0.0018
No	9	3.1	1.5–6.3	27	13.5	8.8–20.0	
Yes	202	96.9	93.7–98.5	209	86.5	80.0–91.2	
**Family structure**	**212**			**229**			0.8512
Single-parent family	118	42.2	33.9–51.0	101	43.3	35.7–51.2	
Two-parent family	94	57.8	49.0–66.1	128	56.7	48.8–64.3	
***Variables for parent/family***							
**Age (years)**	**214**			**235**			0.7249
17–24	23	11.1	5.6–20.8	20	8.5	5.2–13.8	
25–34	99	44.0	35.2–53.1	125	46.9	37.8–56.3	
35–44	78	39.1	30.9–48.1	69	37.3	27.7–48.1	
45–57	14	5.8	3.3–10.0	21	7.2	4.2–11.9	
**Birthplace**	**214**			**236**			0.0670
France	16	5.0	2.6–9.1	1	0.7	0.1–5.0	
Not in France	198	95.0	90.9–97.4	235	99.3	95.0–99.9	
**Parent’s length of time living in France**	**214**			**236**			<0.0001
<10%	18	7.5	3.3–16.1	183	70.3	58.6–79.9	
≥10%	196	92.5	83.9–96.7	53	29.7	20.1–41.4	
**Difficulties in understanding/speaking French (parent)**	**213**			**236**			0.0827
No	158	66.1	56.2–74.7	120	53.4	43.8–62.7	
Yes	55	33.9	25.3–43.8	116	46.6	37.3–56.2	
**Other family members in the greater Paris area**	**214**			**235**			0.0002
No	85	32.4	25.0–40.8	144	60.6	49.8–70.5	
Yes	129	67.6	59.2–75.0	91	39.4	29.5–50.2	
**Educational level**	**211**			**229**			0.0044
None	103	47.3	39.1–55.7	79	31.8	24.0–40.7	
Primary/Middle school	46	25.9	18.3–35.2	47	26.5	17.1–38.7	
High school/University	62	26.8	19.3–35.9	103	41.7	33.9–50.0	
**Monthly household income (euros)**	**204**			**232**			<0.0001
<500	89	31.7	23.8–40.8	152	59.6	51.3–67.3	
≥500	115	68.3	59.2–76.2	80	40.4	32.7–48.7	
**Health insurance**	**214**			**236**			0.0001
None or application in progress	20	9.2	5.2–15.6	65	27.4	21.3–34.5	
Standard health insurance or specific coverage	194	90.8	84.4–94.8	171	72.6	65.5–78.7	
**Number of changes of residence in the previous year**	**211**			**234**			0.0096
0–1	172	79.1	69.9–86.1	145	63.5	54.0–72.1	
≥2	39	20.9	13.9–30.1	89	36.5	27.9–46.0	

**Table 2:** Vaccination coverage at 24 months in the general population (Ile-de-France) and estimated vaccination coverage at 24 months in the children of homeless families (born in or outside France).

Vaccine	General Population, Greater Paris Area	Children Born in France (*n* = 214)		Children Born Outside France (*n* = 236)		Comparison between Children Born in France and Those Born Outside France		
		Estimated Vaccination Coverage %	95%CI	Estimated Vaccination Coverage %	95%CI	Estimated Vaccination Coverage Ratio %	95%CI	*p* Value
**DT-IPV 3 doses**	99.1	98.6	96.1–99.5	47.7	39.4–56.1	2.1	1.7–2.5	<0.0001
**MMR 1 dose**	92.0	90.1	81.6–95.0	33.7	25.4–43.1	2.7	2.0–3.5	<0.0001
**HepB 3 doses**	60.9	73.6	65.4–80.4	26.3	20.6–32.8	2.8	2.2–3.6	<0.0001

DT-IPV: diphtheria, tetanus, and polio; MMR: measles–mumps–rubella; HepB: Hemophilus influenzae type b (Hib)

**Table 3:** Factors associated with insufficiently immunized children for DT-IPV, MMR and HepB based on a multivariate robust Poisson model among children born in France.

	DT-IPV Vaccine (*n* = 211) *			MMR Vaccine (*n* = 157) †			HepB Vaccine (*n* = 159) ‡		
	Adjusted Proportion Ratio	95%CI	*p* Value	Adjusted Proportion Ratio	95%CI	*p* Value	Adjusted Proportion Ratio	95%CI	*p* Value
**Gender**									0.031
Male							1 (ref)		
Female							0.29	0.09–0.89	
**Age (years)**			0.001						
2—5	1 (ref)								
6—9	4.00	1.87–8.59							
≥ 10	2.55	0.87–10.31							
**School attendance**						0.006			<0.001
No				1 (ref)			1 (ref)		
Yes				0.19	0.06–0.61		0.23	0.11–0.49	
**Contact with healthcare at least once in the previous year (child)**			0.042			0.032			
No	1 (ref)			1 (ref)					
Yes	0.30	0.09–0.96		0.18	0.04–0.86				
***Variables for parent/family***									
**Birthplace**			0.001			0.006			
France	1(ref)			1 (ref)					
Outside France	4.93	2.01–12.07		5.54	1.67–18.37				
**Other family members in the greater Paris area**			0.025						
No	1 (ref)								
Yes	0.37	0.15–0.88							
**Health insurance**						0.010			
None or application in progress				1 (ref)					
Standard health insurance or specific coverage				0.36	0.16–0.77				

* significant variables in univariate model: Age, Healthcare contact, Birthplace of parent, Other family members in greater Paris area, Number of changes of domicile in previous year. † significant variables in univariate model: School attendance, Healthcare contact, Birthplace of parent, Family structure, Health insurance. ‡ significant variables in univariate model: Gender, School attendance, Age, Health insurance.

**Table 4:** Factors associated with insufficiently immunized children for DT-IPV, MMR and HepB based on a multivariate robust Poisson model among children not born in France.

	DT-IPV Vaccine (*n* = 234) *			MMR Vaccine (*n* = 227) †			HepB Vaccine (*n* = 232) ‡		
	Adjusted Proportion Ratio	95%CI	*p* Value	Adjusted Proportion Ratio	95%CI	*p* Value	Adjusted Proportion Ratio	95%CI	*p* Value
**Age (years)**			0.014						0.017
2—5	1 (ref)						1 (ref)		
6—9	1.42	1.11–1.82					1.43	1.11–1.82	
≥10	0.99	0.73–1.35					1.30	0.93–1.82	
**Contact with healthcare at least once in the previous year (child)**			0.014			0.035			<0.001
No	1 (ref)			1 (ref)			1 (ref)		
Yes	0.72	0.56–0.93		0.70	0.50–0.97		0.66	0.53–0.81	
***Variables for parent/family***									
**Difficulties with understanding/speaking French (parent)**			<0.001			0.044			
No	1 (ref)			1 (ref)					
Yes	1.47	1.15–1.88		1.51	1.01–2.27				
**Educational level**						0.046			
None				1 (ref)					
Primary/Middle school				0.59	0.36–0.96				
High school/University				0.80	0.59–1.07				
**Monthly household income (euros)**									0.010
<500							1 (ref)		
≥500							0.67	0.49–0.90	
**Health insurance**						0.035			
None or application in progress				1 (ref)					
Standard health insurance or specific coverage				0.58	0.40–0.86				
**Number of changes of residence in the previous year**			0.013			0.015			
0–1	1 (ref)			1 (ref)					
≥2	1.29	1.06–1.58		1.64	1.10–2.45				

* significant variables in the univariate model: Age, Birthplace of parent, School attendance, Healthcare contact, Family structure, Age of parent, Time living in France (Parent), Difficulties understanding/speaking French (Parent), Education level (Parent), Changes of residence in the previous year.† significant variables in the univariate model: Age, School attendance, Healthcare contact, Age (parent), Time living in France (parent), Difficulties understanding/speaking French (parent), Education level (parent), Household income, Health insurance, Number of changes of residence in the previous year.‡ significant variables in univariate model: Age, School attendance, Healthcare contact, Difficulties understanding/speaking French (parent), Other family members in the greater Paris area, Household income, Number of changes of residence in the previous year.
